# Granulocyte-Colony-Stimulating Factor Alters the Proteomic Landscape of the Ventral Tegmental Area

**DOI:** 10.3390/proteomes6040035

**Published:** 2018-09-23

**Authors:** Nicholas L. Mervosh, Rashaun Wilson, Navin Rauniyar, Rebecca S. Hofford, Munir Gunes Kutlu, Erin S. Calipari, TuKiet T. Lam, Drew D. Kiraly

**Affiliations:** 1 Department of Psychiatry, Icahn School of Medicine at Mount Sinai, New York, NY 10029, USA; nicholasmervosh@gmail.com (N.L.M.); rebecca.hofford@mssm.edu (R.S.H.); 2 Fishberg Department of Neuroscience, Icahn School of Medicine at Mount Sinai, New York, NY 10029, USA; 3 Yale/NIDA Neuroproteomics Center, New Haven, CT 06511, USA; rashaun.wilson@yale.edu (R.W.); navin.rauniyar@yale.edu (N.R.); tukiet.lam@yale.edu (T.T.L.); 4 Department of Pharmacology, Vanderbilt Center for Addiction Research, Vanderbilt University School of Medicine, Nashville, TN 37232, USA; gunes.kutlu@vanderbilt.edu (M.G.K.); erin.calipari@vanderbilt.edu (E.S.C.); 5 Department of Molecular Biophysics & Biochemistry, New Haven, CT 06510, USA; 6 Yale MS & Proteomics Resource, New Haven, CT 06510, USA; 7 Seaver Autism Center for Research and Treatment, Icahn School of Medicine at Mount Sinai, New York, NY 10029, USA

**Keywords:** cocaine, addiction, cytokine, neuroimmune, ventral tegmental area

## Abstract

Cocaine addiction is characterized by aberrant plasticity of the mesolimbic dopamine circuit, leading to dysregulation of motivation to seek and take drug. Despite the significant toll that cocaine use disorder exacts on society, there are currently no available pharmacotherapies. We have recently identified granulocyte-colony stimulating factor (G-CSF) as a soluble cytokine that alters the behavioral response to cocaine and which increases dopamine release from the ventral tegmental area (VTA). Despite these known effects on behavior and neurophysiology, the molecular mechanisms by which G-CSF affects brain function are unclear. In this study mice were treated with repeated injections of G-CSF, cocaine or a combination and changes in protein expression in the VTA were examined using an unbiased proteomics approach. Repeated G-CSF treatment resulted in alterations in multiple signaling pathways related to synaptic plasticity and neuronal morphology. While the treatment groups had marked overlap in their effect, injections of cocaine and the combination of cocaine and G-CSF lead to distinct patterns of significantly regulated proteins. These experiments provide valuable information as to the molecular pathways that G-CSF activates in an important limbic brain region and will help to guide further characterization of G-CSF function and evaluation as a possible translational target.

## 1. Introduction

Pathological substance use disorders are a group of recalcitrant, relapsing and remitting conditions that have deleterious effects on the patient, their family, and society at large. While there have been attempts made to mitigate the prevalence of substance abuse disorders, the incidences of illicit substance abuse and misuse has remained steady or increased since 1990 [1], and the economic burden created by substance use disorders is tremendous with a societal cost of over 500 billion dollars per year in the United States alone [2]. Of these conditions, pathological use of psychostimulants such as cocaine and amphetamine account for a significant portion of the morbidity and mortality. However, there are currently no FDA-approved pharmacological treatments for cocaine use disorder [3,4]. Previous drug discovery attempts in this arena have generally failed due to lack of efficacy, intolerable side effects, or both [5,6,7].

In recent years there has been growing interest in the role that neuroimmune interactions play in the development of psychiatric illness, including addictive disorders [8,9,10]. This raises the intriguing possibility that targeting neuroimmune signaling pathways may be a viable translational treatment strategy to reduce the persistence of pathological substance use disorders. Our lab recently discovered granulocyte-colony stimulating factor (G-CSF) as a cytokine that is up-regulated both centrally and peripherally after chronic cocaine treatment [11]. Peripheral injections of G-CSF potentiated the development of locomotor sensitization, conditioned place preference, and self-administration of cocaine, and blockade of G-CSF function in the mesolimbic dopamine system abrogated the formation of conditioned place preference.

While the behavioral effects of G-CSF on cocaine-induced behavioral plasticity are known, the cellular and molecular mechanisms underlying these effects remain to be identified. We have recently found that acute treatment with G-CSF enhances release of dopamine from the ventral tegmental area (VTA) into the nucleus accumbens (NAc) [12]. Previous work has found that the G-CSF receptor is robustly expressed on dopamine expressing neurons of the midbrain [13,14]. G-CSF has been found to be a potent neurotrophic and neuroprotective factor in response to stroke or other insults [15,16,17]. Importantly, G-CSF is also neuroprotective in the midbrain where treatment with G-CSF reduces neuronal death in the MPTP model of Parkinson’s disease [18]. Additionally, within these midbrain neurons, G-CSF has been found to induce activity of the immediate-early gene *Cfos* and acute treatments upregulate tyrosine hydroxylase—the rate limiting step in dopamine synthesis [13]. Moving forward, it will be critical to determine the molecular signaling cascades that control the effects of G-CSF on behavior.

Given the known effects of G-CSF within the midbrain and the importance of the VTA in the development and persistence of substance use disorders [19,20] we characterized the effect of G-CSF and its interaction with cocaine on the proteomic makeup of the VTA. Via an unbiased quantitative proteomics approach, we identified and characterized the regulation pattern of more than two thousand proteins in the VTA. We found that G-CSF treatment on its own regulated many of the same signaling pathways that are regulated by cocaine and induced numerous factors important for neurite and dendritic spine plasticity. Specifically, we found significant regulation of proteins predicted to be downstream from Fragile X mental retardation (FMRP) and mammalian target of rapamycin (mTOR). Additionally, we report multiple intracellular signaling cascades that are differentially regulated by combined cocaine and G-CSF treatment, suggesting future targets for study on the effects of G-CSF on the behavioral response to cocaine.

## 2. Materials and Methods

### 2.1. Animals and Drug Treatments

Male C57BL/6J mice (7 weeks old ~20–25 g; Jackson Laboratories, Bar Harbor, ME, USA) were housed in the animal facilities at Icahn School of Medicine at Mount Sinai. Mice were maintained on a 12:12 h light/dark cycle with lights on at 0700 and lights off at 1900. Mice had food and water available *ad libitum* throughout the experiments. Drug treatments were performed in a 2 × 2 design with the first group receiving phosphate buffered saline vehicle, followed by saline (PBS/Sal), the second group was injected with G-CSF 50 μg/kg (GenScript Biotech, Piscataway, NJ—G-CSF/Sal) followed by saline, the third group was injected with PBS followed by cocaine hydrochloride 7.5 mg/kg (NIDA—PBS/Coc), and the fourth group with both G-CSF and cocaine (G-CSF/Coc). Injections were performed once daily for 7 days and the animals were euthanized 24 h after the final injection. All animals were maintained according to the National Institutes of Health guidelines in Association for Assessment and Accreditation of Laboratory Animal Care accredited facilities. All experimental protocols were approved by the Institutional Animal Care and Use Committee at Mount Sinai.

### 2.2. Protein Preparation

For each mouse the VTA was dissected from fresh tissue on ice using a reference brain atlas and anatomical landmarks to guide dissection. Tissue from each animal was then sonicated into 50 μL of ice-cold RIPA buffer (50 mM Tris [pH 8.0], 1 mM EDTA, 1% Triton X-100, 0.1% sodium deoxycholate, 0.1% SDS, 110 mM NaCl & Halt Protease and Phosphatase Inhibitor Cocktails [Fisher]). Protein concentrations were determined by Bradford colorimetric assay according to manufacturer protocols (Thermo Fisher, Waltham, MA, USA). For these analyses tissue from individual animals was used as distinct data points. There was no pooling of samples between animals other than to make the master mix for cross-assay normalization as described below.

### 2.3. Tandem Mass Tag (TMT) Labeling

**Figure 1:**
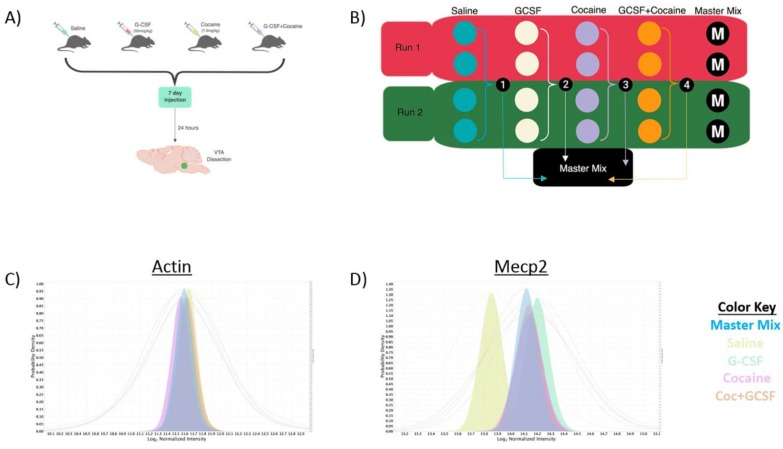
Experimental design and validation. (**A**) Animals were injected with ± G-CSF (50 μg/kg) ± Cocaine (7.5 mg/kg) a 2 × 2 design. Injections were done once daily for 7 days and animals sacrificed 24 h after the final injection and the VTA dissected out for analysis. (**B**) To allow for significant power, two runs of the TMT 10-plex were run with two samples from each group per run (total 4/group) with a mix comprised of an equal amount of each sample run as a Master Mix run to allow normalization between runs. Median intensity values of actin, which was not significantly changed in any group show near complete overlap (**C**) whereas Mecp2 shows increase in all non-saline groups with the expected change in median intensity (**D**).

TMT samples were prepared according to the manufacturer’s instructions. Briefly, 50 µg proteins per condition were reduced by incubating the samples with TCEP (tris(2-carboxyethyl)phosphine) at 55 °C for 1 h and alkylated by incubating with iodoacetamide at room temperature in the dark for 30 min. The proteins were precipitated by the acetone precipitation, resuspended in 25 mM Triethyl ammonium bicarbonate (TEAB) and digested with trypsin at 37 °C overnight. The peptide concentrations of the tryptic digests were measured by Amino Acid Analysis method using a Hitachi L-8900 Amino Acid Analyzer. Equal amount (30 µg) of peptides were labeled with TMT reagents from the TMT-10plex kit (ThermoFisher Scientific). The samples were labeled by distributing them into two experimental groups. Each TMT experimental setup has two TMT tags (126 and 129N) that labeled the two pooled samples, which were created by collecting and combining an equal amount of peptides from each sample. The pooled samples served as a global internal standard for normalizing the data across the two experimental setups and is henceforth referred to as the Master Mix. The remaining 8 TMT reagents in each experimental setup were used for labeling the two biological replicates for each of the four conditions. The TMT labels carried by each sample and the mixing design is shown in Figure 1B. For labeling, the peptides were incubated with TMT reagents for 1 h at room temperature. The labeling reaction was quenched by adding 5% hydroxylamine to the sample and incubating for 15 min. Before combining the labeled samples for mass spectrometry analysis, an aliquot was combined and analyzed by LC-MS/MS to ensure the labeling was complete and also that the mixing generated a ratio of 1. Eventually, all ten labeled samples were combined and fractionated offline by high pH reversed-phase fractionation.

### 2.4. High-pH Reversed-Phase C18 Peptide Fractionation

High-pH reversed-phase C18 peptide fractionation was performed on an ACQUITY UPLC H-class system (Waters Corporation, Milford, MA, USA) on ACQUITY UPLC BEH C18 column, 1.7 µm, 2.1 mm × 50 mm. Elution was performed at a flowrate of 0.4 mL/min using a gradient of mobile phase A (10 mM ammonium acetate) and B (10 mM ammonium acetate in 90% acetonitrile). The gradient extended from 2% to 37% mobile phase B in 17.6 min and then to 75% mobile phase B in another 8.8 min. The collected pooled 10 fractions were dried in a speed-vac centrifuged and reconstituted in buffer A (0.1% formic acid in water); 400 ng digests from each fraction were used for reversed-phase liquid chromatography-tandem mass spectrometry (RP-LC-MS/MS/MS) analysis. 

### 2.5. SPS-MS3 TMT Data Acquisition on an Orbitrap Fusion Tribrid Mass Spectrometer

RP-LC-MS/MS/MS was performed using a nanoACQUITY UPLC system (Waters Corporation, Milford, MA, USA) connected to an Orbitrap Fusion Tribrid (ThermoFisher Scientific, San Jose, CA, USA) mass spectrometer. After injection, samples were loaded into a trapping column (nanoACQUITY UPLC Symmetry C18 Trap column, 180 µm × 20 mm) at a flowrate of 5 µL/min and separated with a C18 column (nanoACQUITY column Peptide BEH C18, 75 µm × 250 mm). The compositions of mobile phases A and B were 0.1% formic acid in water and 0.1% formic acid in acetonitrile, respectively. Peptides were eluted with a gradient extending from 6% to 20% mobile phase B in 120 min and then to 40% mobile phase B in another 50 min at a flowrate of 300 nL/min and a column temperature of 37 °C. The data were acquired with the mass spectrometer operating in a top speed data-dependent mode with multinotch synchronous precursor selection (SPS)-MS3 scanning for TMT tags. The full scan was performed in the range of 380–1580 *m*/*z* at an Orbitrap resolution of 120,000 at 200 *m*/*z* and automatic gain control (AGC) target value of 2 × 10^5^, followed by selection of ions above an intensity threshold of 5000 for collision-induced dissociation (CID)-MS fragmentation in the linear ion trap with collision energy of 35%. The isolation width was set to 1.6 *m*/*z*. The top 10 fragment ions for each peptide MS2 were notched out with an isolation width of 2 *m*/*z* and co-fragmented with higher-energy collision dissociation (HCD) at a collision energy of 65% to produce MS3 scans which were analyzed in the Orbitrap at a resolution of 60,000.

### 2.6. Protein Identification and Quantification

Raw data from the Orbitrap Fusion were processed using Proteome Discoverer software (version 2.1, ThermoFisher Scientific, San Jose, CA, USA). MS2 spectra were searched using Sequest HT which was set up to search against the SwissProt mouse database (downloaded on 06292017). The search criteria included 10 ppm precursor mass tolerance, 0.6 Da fragment mass tolerance, trypsin enzyme and maximum missed cleavage sites of two. Static modification included carbidomethylation (+57.02146 Da) on cysteine and TMT labels (+229.16293 Da) on lysine and peptide N-terminus. Dynamic modifications included oxidation (+15.99492 Da) on methionine, deamidation (+0.98402 Da) on asparagine and glutamine, and acetylation (+42.01057 Da) on protein N-terminus. Peptide spectral match (PSM) error rates were determined using the target-decoy strategy coupled to Percolator modeling of true and false matches [21]. Reporter ions were quantified from MS3 scans using an integration tolerance of 20 ppm and the most confident centroid as the integration method in the Reporter Ions Quantifier node.

### 2.7. Mass Spec Data Analysis

Scaffold Q+ (version Scaffold_4.8.5, Proteome Software Inc., Portland, OR, USA) was used for label-based TMT10-plex quantitation of peptide and protein identifications. Peptide identifications were accepted if they could be established at greater than 95.0% probability by the Scaffold Local FDR algorithm. Protein identifications were accepted if they could be established at greater than 99.0% probability and contained at least 2 identified peptides. Peptide probabilities were calculated by the Scaffold Local FDR algorithm, and protein probabilities were assigned using the Protein Prophet algorithm [22]. Proteins identified with fewer than two peptides were excluded from quantitation. Proteins sharing redundant peptides were grouped into clusters. Normalization was performed iteratively (across samples and spectra) on intensities, as previously described [23]. After setting the minimum dynamic range to 5%, removing spectra that were missing a reference value and those that arose from degenerate peptides that match to more than one protein, the remaining log-transformed spectra were weighted by an adaptive intensity weighting algorithm. Of 71,507 spectra in the experiment, 59,861 (84%) met the threshold criteria and were included in quantitation. Statistical testing was performed using uncorrected Student’s t-test between groups. *p*-values < 0.05 were considered statistically significant. Volcano plots were created using GraphPad Prism version 7 (La Jolla, CA, USA). Pathway analyses to determine specifically regulated pathways were created using Ingenuity Pathway Analysis software from Qiagen. The network diagrams depicted in Figure 3 were created using significantly regulated proteins from our dataset that were predicted to be directly downstream of the hub genes, and then up to 5 genes predicted to be downstream of each of those was added to the outer layer. There were no additional filters applied. Predicted targets downstream from activity-dependent transcription factors was performed using the Enrichr analysis suite (http://amp.pharm.mssm.edu/Enrichr/). Full methodology for the Enrichr analyses is described in detail in the original Chen et al. paper [24]. Heatmaps were created using the freely available Morpheus software from the Broad Institute (https://software.broadinstitute.org/morpheus).

### 2.8. Western Blot Analysis

For Western blot analysis animals were treated identically to those above, and VTA tissue was fresh dissected and frozen on ice until further processing. Samples were thoroughly sonicated into SDS lysis buffer (1% SDS, 50 mM Tris [pH 8.0], 130 mM NaCl, 5 mM EDTA, 50 mM NaF, 1 mM PMSF, protease and phosphatase inhibitor cocktails from ThermoFisher) according to previously published procedures [25]. Sample concentrations were determined using a Bradford colorimetric assay (ThermoFisher) according to manufacturer protocols, and 10μg of protein was run on a 4–12% gradient gel. Proteins were transferred to PVDF membranes using standard techniques. Membranes were blocked using LiCor blocking buffer with TBS based mixed 1:1 with standard TBS for one hour at room temperature. Primary antibodies were incubated with mixing at 4 °C overnight with constant agitation. Primary antibodies used were tyrosine hydroxylase (AbCam #ab112, 1:1000), Mecp2 (Cell Signaling #3456, 1:1000) & actin (Cell Signaling #3700, 1:10,000). Membranes were washed with TBS + Tween-20 before incubation with secondary antibodies raised against the appropriate species (LiCor, 1:10,000) for one hour at room temperature. Membranes were then washed with TBS + Tween-20, rinsed with TBS without Tween, and imaged using a LiCor Fluorescent imager. Image quantification was performed using freely available ImageJ software. Representative images shown in Figure 8 were flipped horizontally to achieve representative bands in the correct order but were not otherwise altered or retouched.

## 3. Results

### 3.1. Experimental Design

We have previously demonstrated that peripheral injections of G-CSF alter gene expression in the NAc in response to cocaine [11]. More recently, we have identified G-CSF as a potent regulator of dopamine release from the VTA into the NAc [12]. These data lead us to the hypothesis that G-CSF may be inducing changes in VTA function that lead to downstream alterations in neuronal responsiveness in the NAc. To assess the effects of G-CSF alone and in combination with cocaine a 2 × 2 experimental design was utilized in which animals were being injected with vehicle, G-CSF (50 μg/kg), cocaine (7.5 mg/kg), or both—with the appropriate additional vehicle controls (Figure 1A). Animals received 7 daily injections as this treatment paradigm leads to significant alterations of important synaptic plasticity pathways and protein changes [26].

To allow for sufficient power to detect protein changes in a complex mixture using this 2 × 2 experimental design, two parallel runs were performed utilizing the TMT-10-plex labeling method as described in the Methods section. To allow for quantitative comparisons between the two runs we pooled an equal amount of each of the experimental samples and ran it in duplicate as the “Master Mix” in each run (Figure 1B). This allowed for a standard for normalizing protein expression between runs and allowed for an N of 4 for each experimental group in the discovery proteomics analysis. Figure 1C,D provides examples of median intensity plots for a regulated (MeCP2, Figure 1D) and non-regulated (Actin, Figure 1C) protein. In Figure 1C the median intensity values for actin-derived peptides are presented with the colored-in peaks representing the median Log2 normalized intensity, and the corresponding lines representing the full range. All groups including the master mix show alignment of their median intensities. In Figure 1D we provide an example of a protein that was shown to be up-regulated relative to Saline in all other treatment groups, Mecp2. The median intensities of the other treatment groups are increased relative to that of Saline. Additionally, within this group the median intensity of the Master Mix is shifted towards the up-regulated groups but is somewhat downshifted compared to the three experimental conditions, suggesting that the lower levels of Mecp2 in the saline samples caused a shift in the Master Mix graph, as would be expected.

### 3.2. Proteomic Effects of G-CSF in the VTA

**Figure 2:**
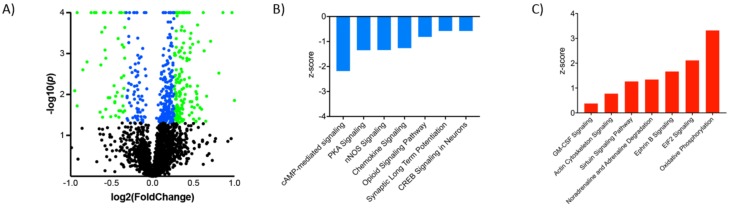
G-CSF regulated proteins and signaling pathways. (**A**) Volcano plot demonstrating proteins in the G-CSF group relative to saline controls with Log2 Fold change on the *x*-axis and Log10 *p* value on the *y*-axis. Proteins that were significantly changed with a nominal *p* value of <0.05 are represented by blue dots, and those with a ±20% change and a *p* < 0.05 are represented by green dots. Ingenuity pathway analysis demonstrated that amongst the significantly regulated proteins there were multiple canonical signaling pathways that were found to be down-regulated (**B**) as well as up-regulated (**C**) relative to saline controls.

For our initial analyses we queried the effects of chronic G-CSF alone on the VTA proteome. While the G-CSF receptor has been shown to be robustly expressed in the midbrain [27,28], there has not yet been a detailed molecular analysis of the effects of chronically increased G-CSF signaling. Figure 2A is a volcano plot of the fold-change and *p*-value of regulation for each protein that was detected in the proteomics analysis. There were 2353 reliably detected proteins, 475 met a threshold of *p* < 0.05 when the normalized mean intensity of detected peptides was compared to those from the saline group (colored dots on volcano plot). Of these 475, we found that 121 were down-regulated and 354 were up-regulated, suggesting that repeated treatment with G-CSF was more likely to upregulate protein networks in the VTA. To look more stringently at proteins that were regulated by repeated G-CSF, we identified proteins that were up or down-regulated by more than 20%. By applying this criterion, we identified 184 proteins 51 were down-regulated and 153 were up-regulated (green dots on volcano plot). A full list of proteins significantly regulated by G-CSF with corresponding fold-change information and *p* values is available as Table S1.

It should be noted that the primary purpose of these experiments was to gain further insight into the effect of G-CSF on the proteomic landscape of the VTA, and to identify important signaling networks for future more mechanistic studies into the effects of G-CSF in the brain. Given this, and that our study was not powered to allow for statistical correction of multiple tests, uncorrected *p* values were utilized in this figure and throughout the manuscript. Additionally, all proteins that were found to be significantly regulated were included in subsequent pathway and network analysis, regardless of the fold change. While this methodology may bias reported results towards an increased number of false positives, we feel that it is appropriate for a discovery analysis such as this one.

To provide a context for how these large-scale protein changes induced by G-CSF might be affecting neuronal function in the VTA we analyzed the subset of proteins found to be significantly regulated to look for changes in intracellular signaling networks. Analysis of canonical signaling pathways identified multiple that were significantly up or down-regulated (Table 1 and Figure 2B,C). Among the seven most significantly down-regulated pathways (Figure 2B), there were multiple that relate to signaling downstream of cyclic-adenosine monophosphate (cAMP)—a second-messenger signaling system heavily implicated in response to drugs of abuse [20]. We see down-regulation specifically of the cAMP-mediated signaling pathway, the protein kinase A (PKA) signaling pathway which is downstream of activated cAMP, and the CREB1 signaling pathway. When looking at pathways that were significantly upregulated (Figure 2C) we see increases in pathways related to transcriptional and translational control. This includes marked increases in the eukaryotic initiation factor 2 (EIF2) pathway which is critical for the initiation of translation from mRNA to protein [29]. Furthermore, sirtuin and granulocyte-macrophage colony stimulating factor (GM-CSF) are also increased. Sirtuins are a class of histone deacetylase enzymes and changes in their function have previously been shown to be important for behavioral response to cocaine and opiates [30,31]. GM-CSF is another colony stimulating factor molecule that shares some signaling pathways with G-CSF, and this increase in this signaling pathway may be due to the overlap in the signaling between the two sets of proteins.

**Figure 3:**
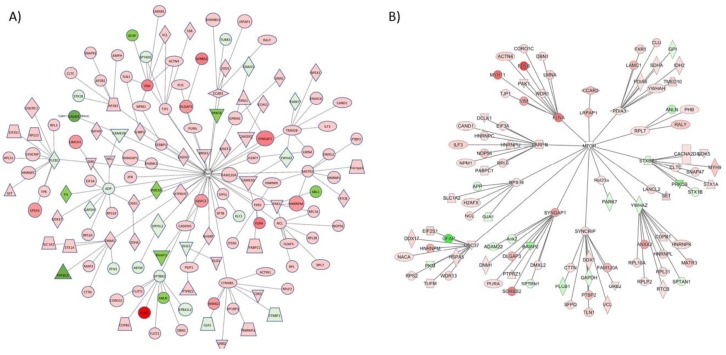
Key upstream regulators of proteins altered by G-CSF. From the proteins identified as significantly altered by repeated G-CSF treatment, we used IPA analysis to identify key upstream regulators. Two of the most robust were FMRP (**A**) and mTOR (**B**). These dendrograms represent all proteins that were significantly changed in this dataset that are predicted to be directly downstream of these regulators, and those that are predicted to be directly downstream of those (two degrees of regulation). Proteins visualized in red are significantly increased, and those in green were significantly decreased.

Given that G-CSF signals through multiple intracellular signaling pathways the data were also analyzed to identify key signaling molecules that might serve as signaling hubs upstream of proteins regulated by G-CSF. These analyses provide information on the specific intracellular signaling networks driven by effects of G-CSF in the VTA. Ingenuity Pathway Analysis (IPA) revealed multiple key regulators predicted to be upstream of proteins regulated by G-CSF as shown in Table 2. The top protein predicted to be an upstream regulator of proteins altered by G-CSF was fragile X mental retardation protein (FMRP), a key protein in translation initiation and the site of the most common mutation seen in Fragile X syndrome. Based on these analyses it was estimated that at least 26 of the proteins that were altered with chronic G-CSF treatment are known to be downstream of FMRP. The data from these samples was used to create a network diagram of all proteins significantly regulated by G-CSF that were predicted to be downstream of FMRP were added. To show the complexity of this network, up to 5 downstream targets of each of the proteins directly downstream of FMRP. This is displayed as a network diagram in Figure 3A. This analysis revealed a total of 157 G-CSF regulated proteins (37 down-regulated and 120 up-regulated—*p* values and fold change values in Table S2) are predicted to be downstream of FMRP.

While FMRP was predicted to be the top upstream regulator of G-CSF-altered signaling networks, it was also noted in our analyses that mTOR was the only protein that was predicted to be a participating regulator in each of the top master regulatory networks identified. Given its apparent broad involvement in those proteins that were regulated by prolonged G-CSF exposure, a network diagram of significantly regulated proteins from our dataset that would be predicted to be downstream of mTOR and its direct effectors was created. This is illustrated in Figure 3B, and from these analyses we see that 101 proteins predicted to be downstream of mTOR are significantly regulated by chronic G-CSF treatment (18 down-regulated, 83 up-regulated—*p* values and fold change values in Table S3).

**Figure 4:**
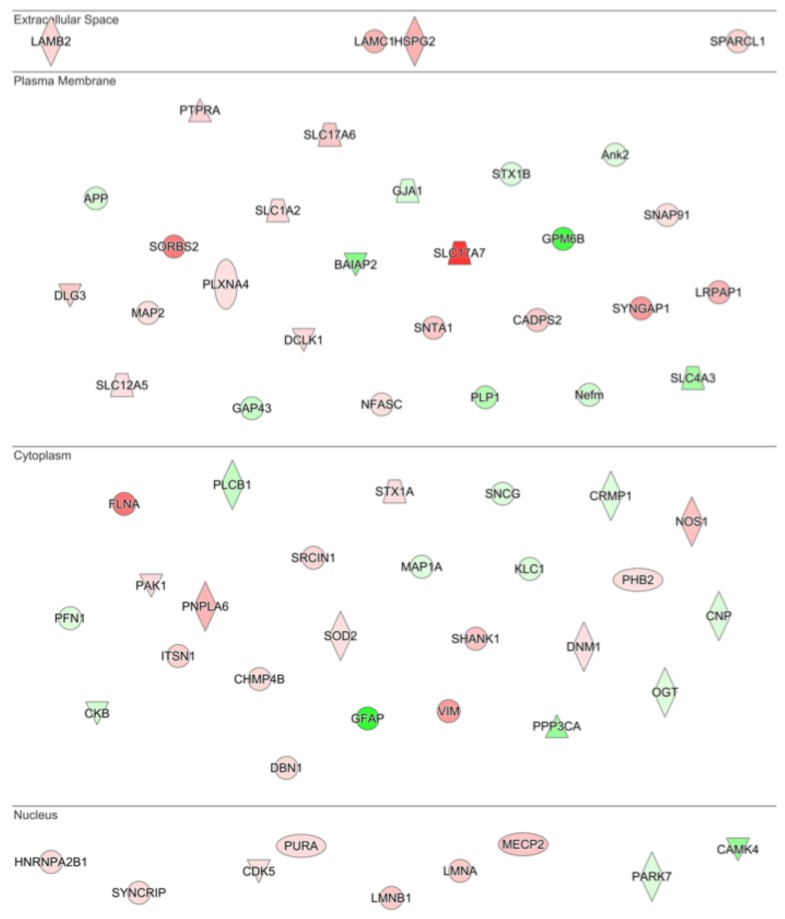
Drivers of neuronal morphology affected by G-CSF. IPA analysis of the most significantly altered cellular functions following G-CSF treatment revealed that proteins involved in altering neuronal morphology were significantly changed. This diagram shows all significantly regulated proteins predicted to be involved in affecting neuronal morphology, and their corresponding predicted subcellular distribution. Proteins visualized in red are significantly increased, and those in green were significantly decreased.

Examination of the most significantly regulated disease and function changes predicted by IPA in the G-CSF treated samples revealed networks related to changes in neuronal morphology, with the most significantly regulated network being “Morphology of Neurons” (*p* = 1.22 × 10^−13^). The significantly regulated proteins belonging to this network are illustrated according to their predicted subcellular distribution in Figure 4—which demonstrates that G-CSF had significant effects on nuclear, cytosolic and cell membrane proteins known to affect the morphological structure of neurons. In sum this network of significantly regulated proteins was comprised of 64 proteins 23 down-regulated and 41 up-regulated (*p* values and fold change values in Table S4).

### 3.3. Interaction Effects of G-CSF & Cocaine in the VTA

**Figure 5:**
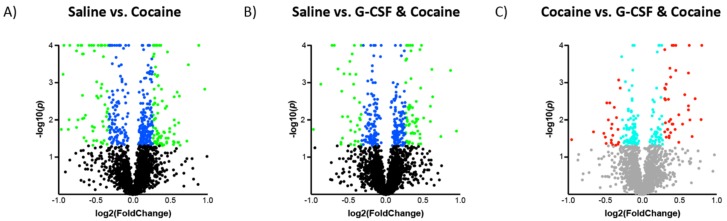
**Changes in VTA protein expression in cocaine-treated groups.** Volcano plots of proteins in the Cocaine (**A**) and Cocaine + G-CSF (**B**) groups relative to saline controls. Log2 Fold change on the *x*-axis and Log10 *p* value on the *y*-axis. Proteins that were significantly changed with a nominal *p* value of <0.05 are represented by blue dots, and those with a ±20% change and a *p* < 0.05 are represented by green dots. (**C**) Demonstrates the changes in the Cocaine + G-CSF group relative to the Cocaine group. Proteins with a nominal *p* value of <0.05 are represented by turquoise dots, and those with a ± 20% change and a *p* < 0.05 are represented by red dots.

Following the analyses of G-CSF treated animals, the effects of cocaine and the combination of cocaine and G-CSF on VTA proteomics were examined (Figure 1A). Cocaine treatment significantly altered 422 with an uncorrected *p* value of <0.05 (Figure 5A—blue dots). Of these, there were 152 that also exhibited a >20% increase or decrease in expression from the saline group (Figure 5A—green dots). Treatment with a combination of G-CSF plus cocaine resulted in 327 proteins that were significantly regulated, 99 of which were increased or decreased by 20% or more compared to saline treatment (Figure 5B). Combination treatment of G-CSF plus cocaine significantly altered 195 proteins, 63 of which were up or down-regulated more than 20% compared to cocaine alone (Figure 5C). A list of all significantly regulated proteins from each pairwise comparison is available as Table S1.

**Figure 6:**
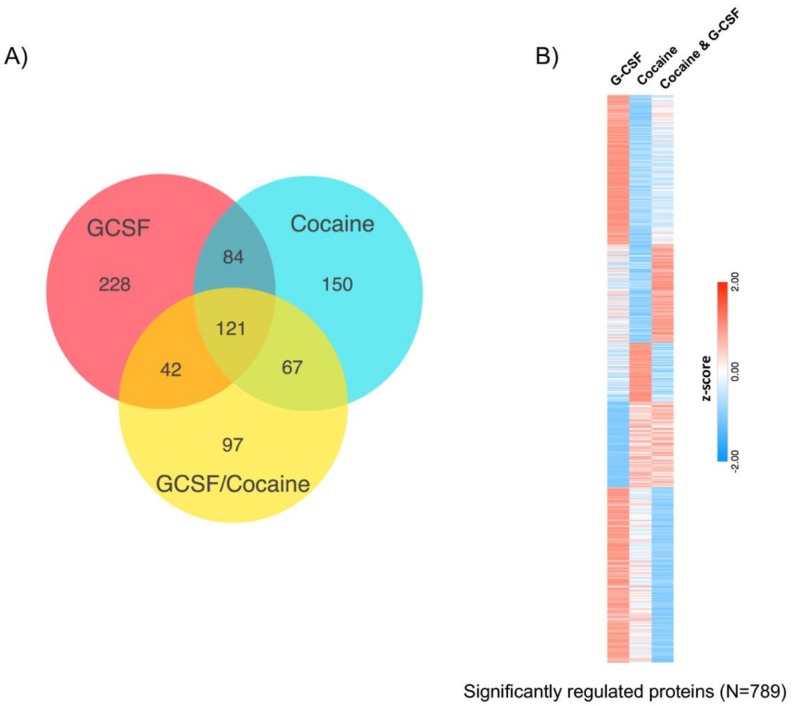
Comparison of significantly-regulated proteins between all treatment groups. (**A**) Venn diagram demonstrating overlap and differences of proteins changed between the three treatment groups relative to saline controls. (**B**) Heatmap visualization of the 789 proteins that were significantly regulated in any treatment group demonstrates clusters of proteins that are differentially affected based on the three treatment groups. K-means clustering (*k* = 5) used to create heatmap of z-scored mean fold-change from saline.

Analysis of all proteins that were regulated compared to saline revealed that there was considerable overlap in changes in protein expression between the groups, but also significant subsets of proteins that were only regulated by one treatment group. Treatment with G-CSF only had the highest number of uniquely regulated proteins (Figure 6A). A breakdown of all proteins in each segment of the Venn diagram is available as Table S5. To further illustrate the differential expression patterns between the treatment groups we measured the mean fold saline expression level of all significantly regulated proteins (N = 789 unique proteins) relative to saline controls. Expression levels were then z-score normalized and sorted using *k*-means clustering (*k* = 5, Figure 6B). Functional characterization of these protein clusters will be the subject of future analyses.

**Figure 7:**
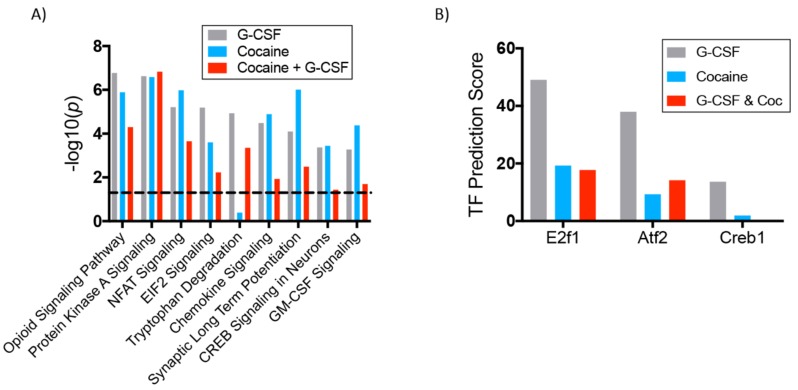
Canonical pathways regulated in all groups and predicted transcription factors of upregulated proteins. (**A**) Ingenuity Pathway Analysis software was used to compare significantly altered canonical signaling pathways amongst all treatment groups. The height of the bars represents the statistical strength of the change but does not represent directionality of change. Directional data available in Table S6. (**B**) Using Enrichr software we identified transcription factors with the highest predicted number of targets in our datasets. This graph demonstrates the calculated transcription factor (TF) prediction score for the three chosen transcription factors. Significantly regulated targets are available in Table S9.

As was done for the G-CSF only group, we also performed IPA analysis of the two cocaine treatment groups to identify canonical signaling pathways that were altered compared to Saline. The *p* values of nine of the most significantly regulated pathways are presented in Figure 7A. These analyses demonstrate that there is indeed a good degree of commonality in the regulated proteins in all three treatment groups compared to the control group. Notably, the tryptophan degradation pathway was significantly regulated in the G-CSF and in the G-CSF + Cocaine groups, but not in the cocaine only group. Full data for these pathways with regulated protein lists and directional z-scores are available as Table S6.

Further pathway analyses was performed utilizing Gene Ontology enrichment analysis (geneontology.org) to assess for specific molecular functions altered in each treatment group relative to Saline controls [32,33,34]. For these analyses, only the upregulated proteins from each treatment group were included. The top 12 significantly regulated molecular processes (as defined by lowest FDR-corrected *p* value) from each treatment group are presented in Table 3. Similar to what was seen with the IPA analyses, we found that 7/12 predicted changes in molecular function were common amongst the three treatment groups. The full list of all significantly changed molecular function pathways is available as Table S7 and the full list of all significantly regulated cellular component pathways is available as Table S8.

Given the substantial number of proteins altered in all treatment groups, analyses were performed to determine which transcription factors were predicted to be affecting the largest number of proteins in the samples. To do this, all of the proteins that were up-regulated relative to saline in each group were uploaded to the Enrichr software package (freely available: http://amp.pharm.mssm.edu/Enrichr/). Using inputs from an exhaustive list of published studies, this software predicts the transcription factors most likely to be upstream of regulated proteins and provides a transcription factor prediction score [24]. Based on this, the transcription factors likely to be responsible for the most changes in each group were identified. For these analyses focus was placed on two transcription factors that achieved statistically significant prediction value for each of the three treatment groups, as well as CREB1 which was significantly regulated only in the G-CSF group, but which has been broadly implicated in the neurobiology of addiction [35,36]. These analyses predicted the E2F1 transcription factor to be the strongest regulator of proteins in the G-CSF group, but is also a significant driver of transcription in the other two experimental groups (Figure 7B and Table S9). A similar pattern is seen for both Atf2 and CREB1. While not yet conclusive, these analyses identify potential hub molecules that are driven by G-CSF signaling to induce neuronal and potentially behavioral plasticity.

### 3.4. Protein Validation

Due to the relatively small sample size (N = 4) of each of the treatment groups and the relatively large number of proteins defined as significantly regulated by various treatments, we performed experiments to validate the scale and directionality of change of some key regulated proteins. For these analyses we chose tyrosine hydroxylase, the rate limiting enzyme in dopamine synthesis, and a protein predicted to be significantly decreased in all three treatment groups. We additionally examined changes in Mecp2, a methyl-DNA binding protein that has been shown to be important in numerous aspects of neuronal and behavioral response to cocaine [37,38], and which was predicted to be increased in all three treatment groups in our mass spec analyses. For these experiments animals received the same treatments as in Figure 1 and protein levels in the VTA were examined with quantitative Western blot analysis.

**Figure 8:**
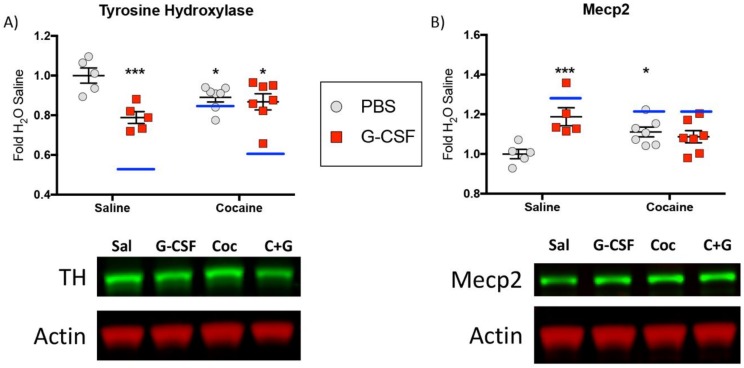
Western blot validations—To validate proteins identified as changed by mass spectrometry additional Western blot analysis of similarly treated tissue was performed. Graphical fold-change from saline control is shown for tyrosine hydroxylase (**A**) and Mecp2 (**B**) with corresponding representative images shown below. Blue lines on the graph represent the fold-change from saline that was seen in each of the treatment groups with mass spec analysis.

Analysis of tyrosine hydroxylase levels demonstrated changes similar in magnitude to those that were reported with the initial analyses. Two-way ANOVA demonstrated a main effect of G-CSF (F_(1,20)_ = 11.63; *p* = 0.003) and a significant G-CSF x cocaine interaction (F_(1,20)_ = 7.612; *p* = 0.012) but no main effect of cocaine (F_(1,20)_ = 0.202; *p* = 0.66). Post-hoc analyses (Fisher’s LSD) demonstrated significant differences with all treatment groups compared to the Saline controls (Figure 8A—asterisks). To compare the results from the Western blots to the mass spec data from above, the fold-change from saline for all groups is marked on the graphs with a blue line. While the magnitude of the changes were not identical, they were quite similar and all in the same direction. A similar pattern for Mecp2 was also seen. We found a main effect of G-CSF (F_(1,20)_ = 6.707; *p* = 0.018) and a significant Cocaine x G-CSF interaction (F_(1,20)_ = 11.19; *p* = 0.003), but no main effect of cocaine (F_(1,20)_ = 0.02; *p* = 0.88). Post-hoc testing demonstrated significant differences between Saline and G-CSF and Saline and Cocaine (Figure 8B) 

## 4. Discussion

We have recently identified G-CSF as a key mediator of neuronal and behavioral plasticity in response to cocaine [11]. In this manuscript an unbiased proteomics analysis is employed to identify protein changes induced in the VTA by G-CSF, both on its own and in combination with cocaine. In our original studies G-CSF signaling in the NAc was found to play a key role in the behavioral effects of G-CSF. Given that dopamine release from VTA terminals in the NAc is a crucial substrate of reward learning and the attribution of salience to rewarding stimuli, understanding changes in protein expression in the VTA is critical for understanding the neuroplasticity that occurs in response to drugs of abuse. Additionally, since the publication of our initial study we found that peripheral injections of G-CSF are capable of modulating dopamine signaling by enhancing release from VTA terminals in the NAc [12]. Given this and the fact that G-CSF receptors are densely expressed in the VTA [28] lead to these proteomic analyses of the VTA.

Review of the literature demonstrates that the exact intracellular signaling mechanisms of G-CSF in the brain are not fully clear and may be complex. G-CSF treatment has variously been shown to induce activity of the Jak-Stat, Erk, and CREB1 signaling cascades among others [28,39,40,41,42]. These results demonstrate that treatment with G-CSF decreased signaling in the CREB1 transcription factor signaling cascades, as well as the cAMP and PKA pathways which are well known to be upstream of CREB1 (Figure 2B) [43]. Increased expression of CREB1 in the NAc and in subregions of the VTA has been shown to decrease cocaine reward, and inhibition of CREB1 in these regions has been shown to enhance reward in a region-specific manner [35,44]. Analysis of significantly upregulated proteins in the G-CSF treatment group found that CREB1 was predicted to be one of the transcription factors driving gene expression (Figure 7). This apparent discrepancy in Figure 2 and Figure 7 may be due to the fact that the IPA analysis looks at networks of proteins based on literature review, while the Enrichr software looks only at those proteins predicted to be directly downstream of the transcription factor. Since G-CSF enhances cocaine intake and place preference and alters CREB1-related signaling, it is possible that the behavioral effects of G-CSF are at least partially mediated through the CREB1 pathway.

We also observed regulation of proteins related to the maintenance of synapses and other cell-cell contacts in our G-CSF treated groups (Figure 2B and Figure 4). This is of particular interest as numerous studies have demonstrated that changes in synapse density are induced by cocaine and are important for the behavioral response to drugs of abuse [45,46]. While most of these studies have focused on the NAc, there is also evidence for synaptic remodeling in the VTA [47,48]. These findings raise the possibility that G-CSF may participate in neurite remodeling, and may prime animals for further changes in synaptic structure in response to cocaine, thus leading to the potentiation of behavioral response induced by G-CSF [11].

The G-CSF-treated animals displayed significant changes in signaling cascades that are related to initiation of mRNA translation. IPA analyses predicted that one of the most up-regulated canonical signaling pathways is the eukaryotic initiation factor 2 (EIF2) pathway which is a critical mediator of protein translation initiation and has been implicated in synaptic plasticity and memory [49] (Figure 2C). Interestingly, EIF2 signaling has been shown to be inhibited by PKA signaling which is found to be decreased in our G-CSF-treated animals (Figure 2B). EIF2 is also known to be activated by the mTOR pathway which was predicted to be a key upstream regulator of the altered proteins in our dataset [50] (Figure 3B). Indeed, the two most highly predicted upstream regulators, mTOR and FMRP (Figure 3), have been shown to be critical regulators of translation of synaptic mRNAs and play key roles in synaptic plasticity [51].

There is a growing literature demonstrating the importance of regulators of synaptic translation regulators in the neuronal and behavioral plasticity in response to cocaine. Recently, an elegant study by the Wolf lab demonstrated increased protein translation during cue-induced drug seeking, and inhibition of mTOR or EIF2 could significantly attenuate cocaine seeking [52]. Studies of FMRP have shown that it is also critical for the rewarding effects of cocaine and changes in synapse structure in response to cocaine [53]. A number of studies have found roles for mTOR-mediated intracellular signaling cascades in NAc in response to cocaine [54,55,56]. Behaviorally it has been demonstrated that inhibition of mTOR with rapamycin can reduce locomotor sensitization, conditioned place preference, and cocaine seeking [57,58,59]. The role of mTOR in the VTA was recently interrogated by Liu and colleagues who found that deletion of mTOR reduced VTA dopamine release and decreased conditioned place preference for cocaine [60].

When examining the number of proteins that were significantly altered between the different treatment groups, it was found that treatment with G-CSF alone leads to changes in the largest number of proteins (Figure 5 and Figure 6). This may be due to the fact that activation of the G-CSF receptor has been coupled to direct activation of transcription factors [39,40,61]. In contrast, cocaine directly leads to changes in multiple neurotransmitter systems, but its effects on gene expression are tightly coupled with context and behavior [62,63,64]. It is interesting that the combination of G-CSF and cocaine lead to the smallest number of regulated proteins of the three treatment groups (Figure 5B). This suggests the possibility that there are interactions between signaling pathways after G-CSF and cocaine in the two that temper changes in protein expression in the VTA.

One of the more surprising findings from these studies was the similarity in changes between treatment groups. Pathways that were regulated by G-CSF, Cocaine, or the combination were largely the same (Figure 7A and Table 3) despite some differences. Given that G-CSF enhances the behavioral effects of cocaine [11] and enhances dopamine release from the VTA [12] one might have suspected that the effects of G-CSF and cocaine on protein expression in the VTA would have been additive. Comparisons of levels of proteins relative to Saline revealed only 42 proteins in which Saline <G-CSF <Cocaine <G-CSF + Cocaine and 107 in which Saline > G-CSF > Cocaine > G-CSF + Cocaine (Table S10). This raises the possibility that the behavioral and physiological responses potentiated by G-CSF may be owing in part to this smaller subset of proteins, or, more likely, that the changes induced by G-CSF are complex and dependent on the function and response of multiple brain regions. Further examination of these clusters of regulated proteins will be important for understanding interactions between G-CSF and cocaine.

While these results have provided new and interesting findings related to the effects of G-CSF and cocaine on proteomic expression in the midbrain, there are important caveats to their interpretation. This study was designed as a discovery analysis to identify G-CSF and cocaine interactions in a 2 × 2 design, and while this allowed us to investigate effects and interactions it lead to a study with low power in terms of sample size (N = 4/group). While we were able to perform successful Western blot validation of several regulated targets (Figure 8) the low sample size and decision not to correct *p* values leads to a high likelihood that some of the reported changes are indeed false positives. Additionally, while the use of network and pathway analyses (IPA, GO, Enrichr) are very useful for the identification of potentially regulated pathways, it is important to note that none of these software packages are built on a comprehensive review of the entire scientific knowledge base, but rather large cross-sections of data that are available to be mined. Additionally, most of these software packages pool data across tissues to increase statistical power in the analyses. While this has utility, it is important to note that regulation of intracellular pathways in other tissues, or even in other brain regions, is likely to be different from that seen in the VTA and has the potential to lead to spurious conclusions.

In sum, we have identified G-CSF as a neuroimmune factor that significantly influences the behavioral and neuronal response to cocaine [11]. While this initial study established the possibility that G-CSF may be a translationally-relevant target for the treatment of cocaine abuse, there remains much to be done to establish its mechanism of action in the brain. Here we present an unbiased proteomic analysis of the VTA animals treated with G-CSF, cocaine, or both. This study identified key intracellular signaling pathways that are altered by systemic G-CSF treatment and lays the groundwork for future mechanistic studies into the effects of G-CSF in brain reward structures.

## Figures and Tables

**Table 1:** Canonical signaling pathways altered by G-CSF treatment. Ingenuity pathway analysis of proteins significantly altered by repeated G-CSF treatment reveals multiple signaling networks that are up and downregulated.

	Ingenuity Canonical Pathways	−log(*p*-value)	z-score
**Downregulated**	cAMP-mediated signaling	2.95	−2.183
	Protein Kinase A Signaling	6.62	−1.347
	nNOS Signaling in Neurons	3.2	−1.342
	Chemokine Signaling	4.48	−1.265
	Opioid Signaling Pathway	6.77	−0.816
	Synaptic Long-Term Potentiation	4.1	−0.577
	CREB Signaling in Neurons	3.37	−0.577
	RhoA Signaling	2.56	−0.333
	Dopamine-DARPP32 in cAMP Signaling	2.83	−0.277
**Upregulated**	Oxidative Phosphorylation	3.78	3.317
	EIF2 Signaling	5.19	2.111
	Ephrin B Signaling	3.24	1.667
	Noradrenaline/Adrenaline Degradation	2.87	1.342
	Sirtuin Signaling Pathway	3.07	1.265
	Tryptophan Degradation	4.93	0.816
	Actin Cytoskeleton Signaling	3.15	0.775
	Thrombin Signaling	3.4	0.535
	GM-CSF Signaling	3.28	0.378
	14-3-3-mediated Signaling	3.93	0.333
	Neuropathic Pain Signaling in Dorsal Horn Neurons	3.07	0.302
	Calcium Signaling	3.46	0.277

**Table 2:** Predicted upstream regulators of G-CSF affected proteins. Data from Ingenuity Pathway Analysis predicting the regulator genes with the greatest influence on significantly regulated proteins from animals treated with daily G-CSF.

Master Regulator	Molecule Type	Participating Regulators	Activation z-score	*p*-Value	Direct Targets
FMR1	Translation Regulator	Akt1, FMR1, MAPT, **MTOR**	1.4	7.83 × 10^−14^	26
MMP9	Peptidase	AKT1, FMR1, GRIN1, MAPT, MMP9, **MTOR**	−1.8	1.51 × 10^−13^	25
CDK5	Kinase	CDK5, FMR1, MAP2, MAP2K1, MAPK1, MAPT, **MTOR**, RPS6KB1, STAT3, TRPV1	−0.78	1.49 × 10^−10^	25
SLC6A3	Transporter	CDK5, GSK3B, MAP2, MAP2K1, MAPK10, MAPT, **MTOR**, RPS6KB1, SLC6A3	1.09	3.76 × 10^−10^	21
EGR1	Transcription Regulator	CDK5, EGR1, GSK3B, MAP2, MAP2K1, MAPK10, MAPT, **MTOR**, RPS6KB1	−1.34	1.05 × 10^−9^	20

**Table 3:** Gene Ontology analysis of significantly regulated molecular functions in each treatment group. Date representing the top 12 significantly regulated molecular functions from each treatment group sorted from smallest to largest FDR-corrected *p* value. Only proteins significantly upregulated relative to saline were included in these analyses. Bolded GO terms are those that were significantly regulated in all three treatment groups.

	GO Molecular Function Complete	REFLIST (22262)	Upload Match	Upload Expected	Upload +/−	Fold Enrichment	Raw *p*-Value	FDR
**G-CSF vs. Saline**	**binding (GO:0005488)**	13001	296	200.9	+	1.47	2.49 × 10^−28^	1.13 × 10^−24^
	**RNA binding (GO:0003723)**	1019	73	15.75	+	4.64	2.86 × 10^−27^	6.48 × 10^−24^
	**protein binding (GO:0005515)**	8900	227	137.53	+	1.65	6.02 × 10^−22^	9.09 × 10^−19^
	**heterocyclic compound binding (GO:1901363)**	4917	152	75.98	+	2	1.82 × 10^−19^	2.07 × 10^−16^
	**organic cyclic compound binding (GO:0097159)**	5006	152	77.35	+	1.96	1.08 × 10^−18^	9.77 × 10^−16^
	mRNA binding (GO:0003729)	243	28	3.75	+	7.46	1.56 × 10^−15^	1.18 × 10^−12^
	protein-containing complex binding (GO:0044877)	1163	58	17.97	+	3.23	9.27 × 10^−15^	6.01 × 10^−12^
	enzyme binding (GO:0019899)	2260	84	34.92	+	2.41	7.23 × 10^−14^	4.10 × 10^−11^
	**signaling receptor activity (GO:0038023)**	2271	2	35.09	−	0.06	1.56 × 10^−13^	7.87 × 10^−11^
	structural molecule activity (GO:0005198)	613	39	9.47	+	4.12	3.37 × 10^−13^	1.53 × 10^−10^
	**molecular transducer activity (GO:0060089)**	2324	3	35.91	−	0.08	1.18 × 10^−12^	4.88 × 10^−10^
	transmembrane signaling receptor activity (GO:0004888)	2082	2	32.17	−	0.06	3.02 × 10^−12^	1.14 × 10^−9^
**Coc. vs. Saline**	***protein binding (GO:0005515)***	*8900*	*170*	*96.35*	*+*	*1.76*	*2.40 × 10^−21^*	*1.09 × 10^−17^*
	***binding (GO:0005488)***	*13001*	*206*	*140.74*	*+*	*1.46*	*2.12 × 10^−19^*	*4.82 × 10^−16^*
	*structural molecule activity (GO:0005198)*	*613*	*32*	*6.64*	*+*	*4.82*	*5.44 × 10^−13^*	*8.23 × 10^−10^*
	***RNA binding (GO:0003723)***	*1019*	*41*	*11.03*	*+*	*3.72*	*7.98 × 10^−13^*	*9.05 × 10^−10^*
	***organic cyclic compound binding (GO:0097159)***	*5006*	*100*	*54.19*	*+*	*1.85*	*6.20 × 10^−13^*	*5.62 × 10^−8^*
	***heterocyclic compound binding (GO:1901363)***	*4917*	*98*	*53.23*	*+*	*1.84*	*1.34 × 10^−10^*	*1.02 × 10^−7^*
	***molecular transducer activity (GO:0060089)***	*2324*	*1*	*25.16*	*−*	*0.04*	*1.66 × 10^−10^*	*1.07 × 10^−7^*
	*identical protein binding (GO:0042802)*	*1840*	*52*	*19.92*	*+*	*2.61*	*1.98 × 10^−10^*	*1.12 × 10^−7^*
	*cytoskeletal protein binding (GO:0008092)*	*936*	*35*	*10.13*	*+*	*3.45*	*3.12 × 10^−10^*	*1.57 × 10^−7^*
	***signaling receptor activity (GO:0038023)***	*2271*	*1*	*24.58*	*−*	*0.04*	*4.19 × 10^−10^*	*1.90 × 10^−7^*
	*mRNA binding (GO:0003729)*	*243*	*18*	*2.63*	*+*	*6.84*	*4.91 × 10^−10^*	*2.02 × 10^−7^*
	*actin binding (GO:0003779)*	*411*	*22*	*4.45*	*+*	*4.94*	*1.85 × 10^−9^*	*7.00 × 10^−7^*
**G-CSF + Coc vs. Saline**	**binding (GO:0005488)**	13001	167	108.62	+	1.54	7.88 × 10^−21^	3.57 × 10^−17^
	**protein binding (GO:0005515)**	8900	137	74.36	+	1.84	2.67 × 10^−20^	6.06 × 10^−17^
	identical protein binding (GO:0042802)	1840	46	15.37	+	2.99	1.67 × 10^−11^	2.52 × 10^−8^
	cytoskeletal protein binding (GO:0008092)	936	31	7.82	+	3.96	9.34 × 10^−11^	1.06 × 10^−7^
	**RNA binding (GO:0003723)**	1019	32	8.51	+	3.76	1.65 × 10^−10^	1.50 × 10^−7^
	structural molecule activity (GO:0005198)	613	24	5.12	+	4.69	6.58 × 10^−10^	4.97 × 10^−7^
	actin binding (GO:0003779)	411	19	3.43	+	5.53	3.48 × 10^−9^	2.25 × 10^−6^
	**molecular transducer activity (GO:0060089)**	2324	1	19.42	−	0.05	7.39 × 10^−8^	3.72 × 10^−5^
	**heterocyclic compound binding (GO:1901363)**	4917	74	41.08	+	1.8	6.61 × 10^−8^	3.75 × 10^−5^
	**signaling receptor activity (GO:0038023)**	2271	1	18.97	−	0.05	1.12 × 10^−7^	5.07 × 10^−5^
	**organic cyclic compound binding (GO:0097159)**	5006	74	41.83	+	1.77	1.38 × 10^−7^	5.68 × 10^−5^
	enzyme binding (GO:0019899)	2260	43	18.88	+	2.28	2.95 × 10^−7^	1.11 × 10^−4^

## Supplementary Materials

The following are available online at http://www.mdpi.com/2227-7382/6/4/35/s1, Table S1: All significantly regulated proteins for each pairwise comparison with corresponding p-value and Log2 Fold Change; Table S2: Significantly regulated proteins predicted to be downstream of FMRP; Table S3: Significantly regulated proteins predicted to be downstream of mTOR; Table S4: Significantly regulated proteins predicted to be involved in neuronal morphology; Table S5: Breakdown of all significantly regulated proteins from all 7 portions of the Venn diagram presented in Figure 6; Table S6: Comparisons of significantly regulated canonical signaling pathways for each treatment group as predicted with Ingenuity Pathway Analysis; Table S7: Gene ontology analysis of predicted molecular function of all significantly upregulated proteins in each pairwise comparison; Table S8: Gene ontology analysis of predicted cellular component of all significantly upregulated proteins in each pairwise comparison; Table S9: Predicted transcription factors of upregulated proteins for each pairwise comparison; Table S10: Lists of significantly regulated proteins following the pattern of Saline < G-CSF < Cocaine < G-CSF + Cocaine and Saline > G-CSF > Cocaine > G-CSF + Cocaine.
